# Children’s giving: moral reasoning and moral emotions in the development of donation behaviors

**DOI:** 10.3389/fpsyg.2014.00458

**Published:** 2014-05-23

**Authors:** Sophia F. Ongley, Marta Nola, Tina Malti

**Affiliations:** ^1^Department of Psychology, University of TorontoMississauga, ON, Canada; ^2^Department of Brain and Behavioral Sciences, University of PaviaPavia, Italy

**Keywords:** giving, moral emotions, moral reasoning, donating, childhood

## Abstract

This study investigated the role of moral reasoning and moral emotions (i.e., sympathy and guilt) in the development of young children’s donating behavior (*N* = 160 4- and 8-year-old children). Donating was measured through children’s allocation of resources (i.e., stickers) to needy peers and was framed as a donation to “World Vision.” Children’s sympathy was measured with both self- and primary caregiver-reports and participants reported their anticipation of guilt feelings following actions that violated prosocial moral norms, specifically the failure to help or share. Participants also provided justifications for their anticipated emotions, which were coded as representing moral or non-moral reasoning processes. Children’s moral reasoning emerged as a significant predictor of donating behavior. In addition, results demonstrated significant developmental and gender effects, with 8-year-olds donating significantly more than 4-year-olds and 4-year-old girls making higher value donations than boys of the same age. We discuss donation behaviors within the broader context of giving and highlight the moral developmental antecedents of giving behaviors in childhood.

## INTRODUCTION

Giving is one of the key social behaviors that distinguishes our species from others (Knafo and Plomin, 2006) and fosters care and cooperation in social interactions (Staub, 1979). It takes many forms, from the reciprocal sharing of toys with friends in preschool, to the anonymous donation of money to a charity, to our society’s centralized division and allocation of resources as part of the social welfare system. Because of its roots in early childhood and its importance to large-scale fairness and care (Malti et al., 2012), a rich body of research in psychology has focused on understanding the development and motivation of children’s giving behaviors (Eisenberg et al., 2014). It is often the case, however, that little distinction is drawn between different subtypes of giving. As a result, a lack of conceptual clarity surrounding subtypes of giving behavior exists and many questions remain regarding their potentially distinct affective and cognitive moral antecedents.

Giving behaviors can be differentiated along multiple dimensions, including anonymity (of either the giver or the recipient), the recipient’s level of need, the cost of the giving behavior, and the degree of reciprocity in the relationship between the giver and the recipient. Sharing, as one type of giving, for example, is often examined in research using the dictator game (Kahneman et al., 1986; Gummerum et al., 2010) in which a single player chooses how many (if any) of a set number of items to allocate to an anonymous other. Sharing is completed privately and there is no opportunity for the recipient to respond, retaliate, or form an evaluation of the (non)sharer (Gummerum et al., 2010). In this way, sharing in the dictator game is anonymous, unreciprocated, and costly (the shared items are typically selected so that they are valuable to the giver and there is no opportunity for the allocated items to be returned). Researchers may vary the contextual features of the dictator game, but in the simplest version, there is no explicit need ascribed to the recipient. Donating, like sharing, is also typically anonymous, costly, and unreciprocated. Unlike sharing, however, givers in donation tasks are confronted with potential recipients who exhibit clear need, often on the basis of poverty (Dlugokinski and Firestone, 1973; Rushton and Wheelwright, 1980), injury (Knight et al., 1994), illness (Boe and Ponder, 1981), or disability (Isen and Noonberg, 1979).

Although a number of studies (Eisenberg and Miller, 1987; Malti et al., 2012; Ongley and Malti, 2014) have investigated the relationship between giving behaviors in general and their affective and cognitive moral antecedents, many questions remain about the moral antecedents of distinct giving behaviors, such as sharing and donating. In particular, existing developmental studies have found that the association between giving behaviors and morality differs on the basis of: (1) the type of giving behavior considered (and its dimensions of anonymity, cost, reciprocity, and need of the recipient), (2) the specific aspect of morality measured (e.g., moral reasoning vs. moral emotions, self-evaluative vs. other- oriented moral emotions, and responses to moral transgressions committed by the self or by others), and (3) the measures used in assessing children’s giving and their cognitive and affective moral development (e.g., self-reports, teacher/parent-ratings, anonymous vs. public giving). In the current study, we therefore consider an important distinction between the giving behaviors of sharing and donating: the absence or presence of explicit need. We also investigate the developmental trajectory and affective-moral antecedents specific to donation behaviors of 4- and 8-year-olds. We then discuss our findings on children’s donating in light of previous, related work on sharing, comparing the motivational factors that may be at play in these two distinct types of giving.

In the current study, we chose to examine sympathy, guilt, and moral reasoning as potential motivators of young children’s donation behaviors. Each of these processes has been highlighted in the existing developmental literature as playing an important role in the development of morally relevant, prosocial behavior.

The affective experience of sympathy stems from the apprehension of another’s emotional state and arouses feelings of concern for others (Eisenberg, 2000). A rich body of research has demonstrated that the experience of sympathy may serve to motivate prosocial, other-oriented behavior through concern for others’ wellbeing and the desire to reduce others’ distress (see Eisenberg et al., 2014). Existing research also suggests that the affective experience of sympathy early in childhood may lead to a general tendency to consider the needs of others and the development of norms of fairness and care (Malti et al., 2007, 2012; Knafo et al., 2008; Ongley and Malti, 2014).

In addition to sympathy, the moral emotion of guilt has also been identified as a key affective process in children’s prosocial moral development (Hoffman, 2000; Malti and Latzko, 2012; Malti and Ongley, 2014). While sympathy is considered to be an other-oriented emotion, guilt is oriented towards the self and towards one’s own actions in relation to moral norms (Malti and Ongley, 2014). Guilt has been defined as a painful feeling of regret over wrongdoing (Ferguson and Stegge, 1998) and arises when one acts in violation of one’s own moral standards (Tangney et al., 2007). The experience of guilt feelings reflects the awareness that one has transgressed his or her own internalized moral rules and takes responsibility for these actions (Malti and Ongley, 2014). When one anticipates that feelings of guilt will accompany potential moral rule violations, such as the failure to help or give to those in need, this anticipation of (and desire to avoid) future guilt encourages a commitment to live up to internalized moral standards (Ferguson and Stegge, 1998; Hoffman, 2000; Carlo et al., 2012; Ongley and Malti, 2014). Existing research has suggested that children’s anticipation of guilt is positively related to actions that are consistent with prosocial moral norms such as helping those in need and sharing fairly with others (Chapman et al., 1987; Gummerum et al., 2010; Ongley and Malti, 2014).

The use of moral reasoning to justify actions or resolve moral dilemmas, as compared to reasoning based on sanctions, hedonistic or self-serving considerations, has also been demonstrated to predict specific types of prosocial responding in children, such as sharing, helping, and cooperation (Larrieu and Mussen, 1986; Miller et al., 1996; Stewart and McBride-Chang, 2000; Hinnant et al., 2013). Together, moral reasoning and moral emotions, such as sympathy and guilt, have been theorized to play an important role in the early development of moral action tendencies and the consideration of others’ welfare (Hoffman, 2000; Arsenio, 2014; Malti and Ongley, 2014).

In light of the theoretical perspectives outlined above and specific findings relevant to giving behavior, we made a series of hypotheses regarding the potential associations between children’s donating behavior and sympathy, guilt, and moral reasoning. Based on findings from previous studies showing that sympathy predicts donating in middle childhood (Knight et al., 1994) and other forms of giving (i.e., sharing) in early childhood (Ongley and Malti, 2014), we hypothesized that sympathy would emerge as a significant predictor of donating across our two age groups. We also expected that the anticipation of moral guilt would be positively associated with donating. This hypothesis was drawn from related research demonstrating a positive relationship between guilt and overt prosocial behavior (Malti and Krettenauer, 2013) and between guilt and other forms of giving (i.e., sharing) in young children (Gummerum et al., 2010; Ongley and Malti, 2014). Finally, we predicted that moral reasoning would also emerge as a significant predictor of donating in early childhood. Though existing research has yielded conflicting findings as to the role of moral reasoning in various forms of giving in childhood, there is existing evidence to suggest that moral reasoning is positively associated with donating in middle childhood (Knight et al., 1994) and with costly giving behaviors (i.e., sharing) in early childhood (Eisenberg-Berg and Hand, 1979; Eisenberg et al., 1985; see however, Gummerum et al., 2008).

## MATERIALS AND METHODS

### PARTICIPANTS

The participants in the current study were a community sample of 160 children and their primary caregivers from a suburban area of a major Canadian city. Participants were 78 4-year-olds [*M* age = 4.44 years, SD = 0.27; 38 girls (49%)] and 82 8-year-olds [*M* age = 8.49, SD = 0.24, 43 girls (52%)]. Participating children and their primary caregivers were fluent in English and the majority of primary caregivers were mothers (79%). As a proxy for socioeconomic status (SES), we asked primary caregivers to report their highest level of education. Fifty-five percent of primary caregivers reported that they had completed a university degree, followed in frequency by the completion of a college degree (23%), graduate degree (14%), and high school diploma (8%). As compared to data from the 2006 Census (Statistics Canada, 2007), the education of participants’ primary caregivers is representative of the general education level in the city from which our sample was drawn. The sample for the current study was ethnically diverse. Ethnic backgrounds reported by primary caregivers include Western European (34%), South Asian (14%), Eastern European (11%), East Asian (4%), Caribbean (3%), West and Central Asian (3%), Southeast Asian (3%), African (3%), Central and South American (3%), and other/multiple origins (18%). Four percent of the primary caregivers chose not to report their ethnic background.

### PROCEDURE

Children and their primary caregivers visited the research laboratory once. At the onset of the session, primary caregivers provided written informed consent for their child’s participation and children provided informed verbal consent. Each child was tested independently in a separate room while his or her primary caregiver filled out a questionnaire on the child’s moral and social development and family demographic information. Each session lasted approximately 45 min and consisted of interview questions on moral and social development, nine variations of the dictator game, and a donation task. All sessions were recorded on video. The testers were undergraduate psychology students who had been extensively trained in interview techniques. As pilot testing did not indicate order effects, tasks were administered in a fixed order, with the measurement of self-reported sympathy following the measurement of guilt feelings, moral reasoning, and the dictator game (though the latter is not discussed as part of the current study). The donation task was the final task in the study. All procedures conformed to APA ethical standards for research with children and were approved by the university’s Research Ethics Board.

### MEASURES

#### Donating

Donating was measured through children’s allocation of resources (i.e., stickers) to needy peers and was framed as a donation to “World Vision.” The donation task was adapted from those used in previous research (see Knight et al., 1994). In a task conducted earlier in the experiment as part of a larger study, children participated in nine variations of the dictator game in which they received stickers from the experimenter and decided whether or not to share any number of these with a hypothetical peer. These sharing tasks were conducted privately and with no feedback from the experimenter. For the donation task itself, participants were then left with a number of stickers (ranging from 0 to 54, described below) and they were told that the experimenter was “collecting stickers for poor children.” The experimenter showed the participant a donation box upon which was displayed a World Vision poster composed of the World Vision logo, motto (i.e., “Lend a hand”), and a photograph of three sad and economically disadvantaged children. Participants were then told that poor children would be happy to receive stickers and they were invited to donate any number of stickers they wished. The experimenter clearly stated the participants’ ownership of the stickers and their option to not donate (i.e., “You don’t have to give away any of your stickers if you don’t want to. These are your stickers”). To allow for ostensibly anonymous donations, the experimenter left the room.

The donation measure was scored as the proportion of stickers in each child’s possession at the start of the donation task that was placed in the donation box. Although all participants received the same number of stickers in the dictator game (all participants received 54 stickers in total over 9 variations of the dictator game), participants shared varying proportions of these with hypothetical recipients. Therefore, at the start of the donation task, the number of stickers in participants’ possession ranged from 0 to 54 (see **Table 1**, which displays descriptive statistics by age group for the number of stickers children possessed at the start of the donation task and the proportion of stickers donated). To ensure that children’s donating was not influenced by the number of stickers in their possession at the start of the donation task, we tested the correlation between the number of stickers possessed and the proportion of these stickers donated to World Vision, *r*(157) = -0.34, *p* < 0.001. This resulting negative association indicates that children with fewer stickers in their possession were not influenced away from donating by their relatively small amount of stickers, but instead donated more than their “richer” peers. It is important to note that this negative association between the number of stickers possessed and the proportion of stickers donated is what would be expected, given that the children with fewer stickers in their possession were those who shared more in earlier tasks. To further ensure that there were no systematic associations between the number of stickers in participants’ possession at the start of the donation task and other key variables in the study, we tested for potential correlations between the number of stickers possessed and each of the key study variables (i.e., moral reasoning, child-reported sympathy, caregiver-reported sympathy, and guilt). No significant correlations emerged.

**Table 1:** Descriptive statistics by age group for number of stickers children possessed at start of donation task and proportion of stickers donated.

Age group	Number of stickers in possession			Proportion of stickers donated		
	Minimum	Maximum	*M* (SD)	Minimum	Maximum	*M* (SD)
4-Year-Olds	0	54	31.68 (14.59)	0	1.00	0.27 (0.32)
8-Year-Olds	12	48	28.49 (7.00)	0	1.00	0.53 (0.28)

#### Sympathy

Children’s sympathy was measured using children’s self-reports and ratings by primary caregivers.

***Self-reported sympathy***. Children’s self-reported sympathy was measured with five items from Zhou et al.’s (2003) child-report sympathy scale, which is used widely in research with children (see, for example, Malti et al., 2009; Catherine and Schonert-Reichl, 2011; Ongley and Malti, 2014). A sixth reverse-coded item from Zhou et al.’s (2003) child-report sympathy scale was included in testing (“I don’t feel sorry for other children who are being teased or picked on”), but was excluded from analyses due to low reliability. Participants heard each statement read aloud (e.g., “I often feel sorry for other children who are sad or in trouble”) and after each was asked whether the sentence describes him/her or not, and if so, how strongly. Participants were asked to answer spontaneously and not think too long about their answers. Responses were scored as follows: *this is not like me* was scored as 0, *this is sort of like me* was scored as 1, and *this is really like me* was scored as 2. Cronbach’s α for the child-reported sympathy scale was.69 for 4-year-olds and.68 for 8-year-olds.

***Caregiver-reported sympathy***. Primary caregiver-reports of their child’s sympathy were obtained using all five items from Zhou et al.’s (2003) parent-report sympathy scale (e.g., “My child gets upset when he/she sees another child being hurt”). Primary caregivers responded to the five statements as part of the questionnaire package. Responses were scored as follows: *not at all true* was scored as 1, *often not true* was scored as 2, *somewhat not true* was scored as 3, *somewhat true* was scored as 4, *often true* was scored as 5, and *always true* was scored as 6. Cronbach’s α for the caregiver-reported sympathy scale was. 0.85 and. 0.89 as reported for 4- and 8-year-old children, respectively.

#### Guilt feelings and moral reasoning

To measure children’s anticipation of guilt feelings and moral reasoning (i.e., justifications for anticipated emotions that refer to moral norms or empathic concern for the victim), participants listened and responded to two vignettes depicting moral rule violations (Malti, 2011). The vignettes were adapted from those used in previous research examining the development of moral emotions in the happy-victimizer paradigm (see Malti and Krettenauer, 2013; Arsenio, 2014). Both vignettes represented a situation in which a child has failed to perform a prosocial action, specifically helping or sharing. The two vignettes were read aloud with accompanying illustrations as follows: (1) “Toby’s mom makes two cupcakes, one for Toby, and one for Kevin. Toby decides to eat the two cupcakes and give none to Kevin”; and (2) “One of the boys in Luke’s class was sick when the rest of the class learned a new song that all the students must learn. The boy asks Luke if he could teach him the song but Luke says ‘no’.” The gender of the characters in each vignette was matched to that of the participant and the wording of the vignettes was slightly modified to be appropriate for each age group. After hearing both vignettes, participants were asked to describe how they would feel if they had performed the action in the vignette (i.e., moral emotion) and why they would feel this way (i.e., moral reasoning). These verbal responses were transcribed verbatim by the experimenter. This procedure is consistent with previous research using the happy-victimizer paradigm (Malti et al., 2009; Arsenio, 2014).

***Coding for guilt***. Participants’ first spontaneously mentioned emotion in response to each vignette was coded as anger, fear, sadness, happiness, pride, guilt, disgust, anxiety/worry, embarrassment/shame, neutral, feeling good, feeling bad, describing a psychosomatic complaint, or other. The anticipation of feeling guilty, sad, or bad was coded as representing the anticipation of guilt (see Malti et al., 2009). Other immoral or amoral negative emotions (e.g., anger, fear, or disgust), along with positive emotions and neutral states were coded as not representing guilt. This coding system was based on those used previously in related research (e.g., Malti et al., 2009) and it includes the basic emotional correlates of guilt so that guilt expectancies can be examined in young children who may not be able to explicitly label complex emotions (i.e., guilt) but can already name their basic emotional correlates (Tracy et al., 2005; Malti and Ongley, 2014; Ongley and Malti, 2014). Inter-rater reliability for the coding of guilt was κ = 0.99 based on 15% of the data.

Proportional scores for guilt were created by aggregating the scores from the two vignettes: 0 = no anticipation of guilt in response to either vignette, 0.50 = anticipation of guilt in response to one of the two vignettes, and 1.00 = anticipation of guilt in response to both vignettes. The aggregation of scores was justified as there was a significant association between the guilt scores for the two vignettes, *r*_Φ_(146) =0.24, *p* = 0.004.

***Coding for moral reasoning***. Participants’ justifications for their anticipated emotions were coded as either moral reasons (i.e., those which refer to moral norms and empathic concern for the victim, such as “It is not fair to steal” or “The other child will be sad”) or non-moral reasons (i.e., those which refer to sanctions by an authority, such as “The teacher might find out and get angry,” hedonistic or self-serving reasons, such as “I just like cupcakes so much,” unelaborated reasons, such as “It isn’t nice”), or other, unclassifiable reasons.

Proportional scores for moral reasoning were created by aggregating the scores from the two vignettes: 0 = moral reasoning was not used to justify emotions in response to either vignette, 0.50 = moral reasoning was used to justify emotions in response to one of the two vignettes, and 1.00 = moral reasoning was used to justify emotions in response to both vignettes. The aggregation of scores was justified as there was a significant association between the moral reasoning scores for the two vignettes, *r*_Φ_(128) = 0.27, *p* = 0.002.

## RESULTS

### PRELIMINARY ANALYSES

**Table 2** displays the means and standard deviations of the study variables by age group and gender.

**Table 2:** Means and standard deviations of study variables by age group and gender.

Variable	4-Year-olds (*n* = 78)		8-Year-olds (*n* = 82)	
	Girls *M* (SD)	Boys *M* (SD)	Girls *M* (SD)	Boys *M* (SD)
Donating	0.37 (0.34)^*,a^	0.17 (0.27)^*,A^	0.54 (0.26)^b^	0.52 (0.31)^B^
Child-reported sympathy	0.59 (0.52)^a^	0.63 (0.51)^A^	1.52 (0.42)^*,b^	1.21 (0.48)^*,B^
Caregiver-reported sympathy	4.49 (0.77)^a^	4.59 (0.85)^A^	5.05 (0.50)^*,b^	4.53 (1.04)^*,A^
Guilt	0.61 (0.44)^a^	0.62 (0.38)^A^	0.64 (0.37)^a^	0.56 (0.40)^A^
Moral reasoning	0.03 (0.13)^a^	0.00 (0.00)^A^	0.26 (0.33)^b^	0.17 (0.31)^B^

* Asterisks indicate significant gender differences (*p* < 0.05) within age group.

a,b Different lower case letter superscripts indicate significant age differences (*p* < 0.05) for girls.

A,B Different upper case letter superscripts indicate significant age differences (*p* < 0.05) for boys.

**Table 3** displays the correlations between study variables and between study and control variables (i.e., child age, gender, and primary caregiver’s level of education). As can be seen, donating was positively correlated with child-reported sympathy, moral reasoning, and child age. Donating was also negatively correlated with child gender (gender was dummy coded, girls = 0, boys = 1), with girls donating more than boys. In addition, child-reported sympathy was positively correlated with moral reasoning, child age, and primary caregiver’s level of education. Caregiver-reported sympathy was negatively correlated with primary caregiver’s level of education and children’s use of moral reasoning was positively correlated with their age.

**Table 3:** Correlation matrix of study and control variables.

Variable	1	2	3	4	5	6	7
1. Donating	–						
2. Child-reported sympathy	0.30^***^	–					
3. Caregiver-reported sympathy	0.10	0.12	–		
4. Guilt	0.14^†^	0.04	-0.05	–	
5. Moral reasoning	0.35^***^	0.34^***^	0.04	0.10	–		
6. Child age	0.39^***^	0.62^***^	0.15^†^	0.00	0.36^***^	–	
7. Child gender	-0.18*	-0.14^†^	-0.14^†^	-0.04	-0.14	-0.03	–
8. Primary caregiver’s level of education	0.12	0.18^*^	-0.18^*^	-0.04	0.05	0.12	0.12

*Child age is measured in years. Child gender is dummy-coded (girls = 0, boys = 1).*

† p < 0.10

* p < 0.05

** p < 0.01

*** p < 0.001.

Age and gender differences were also analyzed for each of the central study variables. First, four 2 (age group) × 2 (gender) between-subjects analyses of variance (ANOVAs) were conducted to examine age and gender differences in moral reasoning and in each of the emotion variables. Means and standard deviations of moral reasoning, child-reported sympathy, caregiver-reported sympathy, and guilt for boys and girls within each age group are reported in **Table 2**, as are significant gender differences within each age group and age effects for boys and girls. Next, differences in donating across age groups and gender were examined using a 2 (age group) × 2 (gender) between-subjects ANOVA. Main effects of both age group and gender were found for children’s donating behavior, *F*(1,153) = 28.82, *p* < 0.001, $\eta_{p}^{2}$ = 0.16 and *F*(1,153) = 5.59, *p* = 0.019, $\eta_{p}^{2}$ = 0.04, respectively, with 8-year-olds donating more than 4-year-olds and girls donating more than boys (**Table 2**). Although there was only a marginally significant interaction between age group and gender, *F*(1,153) = 3.66, *p* = 0.058, $\eta_{p}^{2}$ = 0.02, tests of simple effects indicate that gender differences in donating are significant for 4-year-olds only (**Table 2**).

## PREDICTION OF DONATING BEHAVIOR BY SYMPATHY, GUILT, AND MORAL REASONING

To test our hypotheses regarding the predictive effects of sympathy, guilt, and moral reasoning on donating, a hierarchical multiple regression analysis was performed using donation as the dependent variable. As previous research has found associations between giving behavior and age (Benenson et al., 2007; Malti et al., 2012; Ongley and Malti, 2014), gender (Benenson et al., 2007; Leman et al., 2009), and SES (Carlo et al., 2011), we entered child’s age group, gender, and primary caregiver’s level of education as control variables in step 1 of the regression model. Child-reported sympathy, caregiver-reported sympathy, guilt, and moral reasoning were entered in step 2, and interaction terms between all control variables, study variables, and between control and study variables were entered in step 3. All predictor variables were centered at the mean, with the exception of gender and age group. Interaction terms were created by calculating the products of the mean-centered variables (Aiken and West, 1991). In preliminary analyses, we tested all possible interactions but non-significant interaction terms were not retained in the final model. The final model was examined for multicollinearity using the tolerance statistic. Tolerance values for the regression model ranged from 0.40 to 0.98, safely exceeding 0.20, the guideline described by Menard (1995) as the point below which multicollinearity may be biasing a model.

**Table 4** displays the results of the final analysis. Results indicated that donating behavior was predicted by child’s age, moral reasoning, and an interaction between child-reported sympathy and gender, *R*^2^ = 0.26, *F*(8,129) = 5.72, *p* < 0.001. *R*^2^ of 0.26 indicates a large effect size (Cohen, 1988). Thus, older children and children more frequently using moral reasoning donated more. To interpret the interaction between child-reported sympathy and gender, we used the procedure recommended by Aiken and West (1991) and the worksheet created by Dawson (n.d.) for plotting interactions between an unstandardized variable and a binary moderator. We performed *t* tests on two simple slopes, which represented the regression of donating on child-reported sympathy for boys and girls, to determine if they differed significantly from zero. For both genders, simple slopes were evaluated at low and high levels of sympathy. The low and high values of sympathy correspond to the response anchors from Zhou et al.’s (2003) child-report sympathy scale. A mean sympathy score (participant’s average score across 5 items) of 0 indicates weak or no identification with sympathetic statements (“this does not sound like me”) and a mean sympathy score of 2 indicates strong identification with sympathetic statements (“this is really like me”). Neither simple slope was significantly different from zero, with gradients of -0.08 and 0.11, *ns*, and *ns*, for girls and boys, respectively. The simple slopes for girls and boys, were, however, significantly different from each other, with higher levels of sympathy trending towards a slight increase in donation for boys, while no such increase in donation occurred with higher levels of sympathy in girls.

**Table 4:** Hierarchical multiple regression analyses predicting children’s donating behavior.

Predictor	Δ*R*^2^/Δ*F*^2^	β
Step 1	0.17/8.98^***^	
Age group		0.35^***^
Gender		-0.15^†^
Primary caregiver’s level of education		0.10
Step 2	0.07/2.75^*^	
Age group		0.27^**^
Gender		-0.11
Primary caregiver’s level of education		0.12
Child-reported sympathy		0.00
Caregiver-reported sympathy		0.08
Guilt		0.13^†^
Moral reasoning		0.21^*^
Step 3	0.03/5.14^*^	
Age group		0.29^**^
Gender		-0.11
Primary caregiver’s level of education		0.09
Child-reported sympathy		-0.15
Caregiver-reported sympathy		0.11
Guilt		0.14^†^
Moral reasoning		0.20^*^
Child-reported sympathy x gender		0.23^*^
Total *R*^2^	0.26^***^	
*N*	138	

† p < 0.10

* p < 0.05

** p < 0.01

*** p < 0.001.

## DISCUSSION

The present study investigated the development of donating, a specific subtype of giving, in early and middle childhood, as well as the role of affective-moral and cognitive-moral variables as antecedents of donation behaviors. The act of giving can take many forms and previous research has left many questions unanswered regarding the distinctions between subtypes of giving and their moral antecedents in childhood. We believe that it is important to consider whether the factors that motivate children to donate to needy strangers are the same factors that motivate children to share with their peers, and if not, what affective and cognitive processes are important in each. By examining different subtypes of giving, we can enrich our understanding of the factors that motivate children to give in different contexts and this understanding can, in turn, enable researchers, parents, and educators to better understand, foster, and diversify young children’s emerging prosociality.

To test our hypotheses regarding the role of affective-moral and cognitive-moral antecedents of donation behaviors in early and middle childhood, we tested the predictive effects of sympathy, guilt, and moral reasoning on donating. We found that donating was predicted by children’s age, moral reasoning, and an interaction between gender and child-reported sympathy. Existing research on sharing has found that the moral emotions of sympathy and guilt are important motivators of sharing behavior (Gummerum et al., 2010; Malti et al., 2012; Ongley and Malti, 2014). Interestingly, in the present examination of donation behaviors, we did not find that moral emotions played a central role (although guilt was a marginally significant predictor). Although child-reported sympathy was associated with donating at the bivariate level, it did not emerge as a strong predictor of donating when other morally relevant processes (i.e., guilt and moral reasoning) were controlled for. In this case, moral reasoning emerged as a stronger predictor of children’s donating than either sympathy or guilt. This contrast between the current findings on donating and previous studies on sharing suggests that different moral-developmental antecedents may vary in the strength with which they motivate different types of giving. More specifically, the present findings suggest that children’s donation behaviors may be more strongly related to the cognitive process of reasoning about others’ needs while deciding to donate than to the donator’s tendency to experience sympathy and guilt.

Although both donating and sharing behaviors are anonymous, costly, and unreciprocated forms of giving, they differ in the level of need demonstrated by the recipient. In particular, the recipients of donations typically demonstrate clear, explicit need. The findings of the present study suggest that this clear need on the part of donation recipients may be a key factor in this specific type of prosocial behavior. In previous work, researchers have argued that sharing is motivated by moral emotional processes through the affective comprehension of and concern for others’ emotional states (i.e., sympathy) or through the anticipation of one’s own negative affective state after the violation of a moral norm (i.e., guilt; Gummerum et al., 2010; Malti et al., 2012; Ongley and Malti, 2014). Taken together, these results support the importance of moral emotions in children’s development of sharing behaviors. It may be the case that moral reasoning is less important in the motivation of sharing than moral emotions because there is no clear moral imperative to share with those who are not in need. Our results show that in donation behaviors, however, children’s giving is also likely to depend upon a cognitive process in which one attends to and appreciates specific characteristics of the recipient (i.e., explicit need) and the consequences of a decision not to donate. Therefore, higher levels of moral reasoning may produce an increased tendency to donate because children realize that it is their duty towards individuals in need. This mechanism could lead to the important role of moral reasoning in the motivation of donation behaviors, although being sympathetic with a recipient and feelings of guilt upon the transgression of a moral norm maintain relevance in children’s orientation towards others. Nevertheless, other, related interpretations of the current results could consider additional inter-individual differences and their potential role in predicting children’s donation behaviors, such as the role of personality, temperament, the level of children’s knowledge and consideration of conventions and social norms in general, and their theory of mind.

In addition to our main findings, we found developmental and gender differences in moral reasoning, moral emotions, and donating. Gender differences in the current study suggest that school-aged girls report higher levels of sympathy than boys of the same age and caregivers report age-related increases in sympathy for girls only. Higher levels of donating were found in school-aged than in preschool-aged children, and, amongst preschoolers, girls donated more than boys. We also found that both sympathy and moral reasoning increased with age, which is consistent with previous research (for reviews, see Eisenberg et al., 2014; Malti and Ongley, 2014). Although existing studies have yielded conflicting findings regarding young children’s ability to provide moral reasons for specific actions and decisions, the age-related increases in moral reasoning found in the current study support the idea that complex moral reasoning only emerges once children have learned to integrate the often conflicting perspectives of the self and others and have acquired interpretative understanding (Malti et al., 2010; Sokol et al., 2010).

The current study has several limitations. Firstly, we used a cross-sectional design, which does not allow for the examination of intra-individual differences and associations between moral reasoning, moral emotions, and donating across time. Therefore, we recognize that our data are correlational in nature and prevent us from being able to interpret results in terms of causal effects. Future studies on the development of giving behaviors and their cognitive and affective moral antecedents across time would benefit from longitudinal designs. Secondly, although the sample for this study was ethnically diverse and representative of the general socio-economic status of the city from which our sample was recruited, participants’ families were largely from mid- to high-levels of socio-economic status. Thus, findings from the current study cannot be generalized to less advantaged children. As donating involves the recognition of a recipient’s need, less advantaged children may be more likely to empathize with the recipient and their pattern of donations may differ from those of more advantaged children. Future work should include a broad sample of children that represents more diverse levels of socio-economic status. The current study also relied upon responses to a short set of hypothetical scenarios to measure guilt and moral reasoning. Future research would benefit from the use of multiple methods of assessing moral emotions and reasoning, including children’s responses to their own experienced moral conflicts, and a more extensive set of scenarios. As multiple variables that were not investigated in this study have been found to be associated with children’s global prosocial behavior, such as theory of mind, inhibitory control, and peer acceptance/rejection (Kochanska et al., 1997; Caputi et al., 2012), they may play also role in children’s donation behaviors. Future work should seek to address this issue by adding measures of children’s socio-cognitive ability (e.g., theory of mind tasks), temperament (e.g., inhibition), and knowledge about (and consideration of) social norms in general to investigations of children’s giving. Finally, two marginal effects in the current study (i.e., the prediction of donation behavior by guilt and the prediction of boys’ donations by self-reported sympathy) suggest that future studies with a larger sample, and thus greater predictive power, may help to clarify the role of moral emotions in donation behaviors.

Despite these limitations, the present study has several notable strengths. Most importantly, this study investigated a relatively unexplored issue – the differentiation between specific types of giving behaviors and their moral-developmental antecedents in childhood. The current study explored this issue in an ethnically diverse sample across two different age groups and demonstrated that children’s donating is motivated by developmental and cognitive moral processes, specifically, children’s use of moral reasoning. These results differ from previous work on sharing, which suggests that sharing is strongly motivated by affective-moral processes such as the moral emotions of sympathy and guilt. As a result, the present study provides valuable contributions to our knowledge about the development of children’s giving behaviors and why children are motivated to give costly resources to others.

## Conflict of Interest Statement

The authors declare that the research was conducted in the absence of any commercial or financial relationships that could be construed as a potential conflict of interest.
